# Glucagon-like peptide-1 receptor agonists and heart failure in type 2 diabetes: systematic review and meta-analysis of randomized and observational studies

**DOI:** 10.1186/s12872-016-0260-0

**Published:** 2016-05-11

**Authors:** Ling Li, Sheyu Li, Jiali Liu, Ke Deng, Jason W. Busse, Per Olav Vandvik, Evelyn Wong, Zahra N. Sohani, Malgorzata M. Bala, Lorena P. Rios, German Malaga, Shanil Ebrahim, Jiantong Shen, Longhao Zhang, Pujing Zhao, Qunfei Chen, Yingqiang Wang, Gordon H. Guyatt, Xin Sun

**Affiliations:** Chinese Evidence-based Medicine Center, West China Hospital, Sichuan University, 37 GuoXue Xiang, Chengdu, 610041 Sichuan China; Department of Endocrinology and Metabolism, West China Hospital, Sichuan University, Chengdu, Sichuan China; West China school of Pharmacy, Sichuan University, Chengdu, Sichuan China; Department of Clinical Epidemiology and Biostatistics, McMaster University, Hamilton, ON Canada; Department of Anesthesia, McMaster University, Hamilton, ON Canada; The Michael G. DeGroote Institute for Pain Research and Care, McMaster University, Hamilton, ON Canada; Norwegian Knowledge Centre for the Health Services, Oslo, Norway; Department of Medicine, Innlandet Hospital Trust, Gjøvik, Norway; Department of Medicine, University of British Columbia, Vancouver, BC Canada; Faculty of Medicine, University of Toronto, 1 King’s College Circle, Toronto, ON Canada; Department of Hygiene and Dietetics, Jagiellonian University Medical College, Krakow, Poland; Systematic Reviews Unit-Polish Cochrane Branch, Jagiellonian University Medical College, Krakow, Poland; Internal Medicine Unit, Hospital Clinico FUSAT, Rancagua, Chile; Department of Medicine, Universidad Peruana Cayetano Heredia, Lima, Peru; Stanford Prevention Research Center, Department of Medicine, Stanford University, Stanford, USA; Department of Anaesthesia & Pain Medicine, The Hospital for Sick Children, Toronto, ON Canada; The Second Hospital of Lanzhou University, Lanzhou, China; Department of Medical Administration, 363 Hospital, Chengdu, Sichuan China; Department of Medicine, McMaster University, Hamilton, ON Canada

**Keywords:** Glucagon-like peptide-1 receptor, Heart failure, Type 2 diabetes, Systematic review, Meta-analysis

## Abstract

**Background:**

The effect of glucagon-like peptide-1(GLP-1) receptor agonists on heart failure remains uncertain. We therefore conducted a systematic review to assess the possible impact of GLP-1 agonists on heart failure or hospitalization for heart failure in patients with type 2 diabetes.

**Methods:**

We searched MEDLINE, EMBASE, the Cochrane Central Register of Controlled Trials (CENTRAL) and ClinicalTrials.gov to identify randomized controlled trials (RCTs) and observational studies that addressed the effect of GLP-1 receptor agonists in adults with type 2 diabetes, and explicitly reported heart failure or hospitalization for heart failure. Two paired reviewers screened reports, collected data, and assessed the risk of bias. We pooled data from RCTs and observational studies separately, and used the GRADE approach to rate the quality of evidence.

**Results:**

We identified 25 studies that were eligible for our review; 21 RCTs (*n* = 18,270) and 4 observational studies (*n* = 111,029). Low quality evidence from 20 RCTs suggested, if anything, a lower incidence of heart failure between GLP-1 agonists versus control (17/7,441 vs. 19/4,317; odds ratio (OR) 0.62, 95 % confidence interval (CI) 0.31 to 1.22; risk difference (RD) 19 fewer, 95 % CI 34 fewer to 11 more per 1000 over 5 years). Three cohort studies comparing GLP-1 agonists to alternative agents provided very low quality evidence that GLP-1 agonists do not increase the incidence of heart failure. One RCT provided moderate quality evidence that GLP-1 agonists were not associated with hospitalization for heart failure (lixisenatide vs placebo: 122/3,034 vs. 127/3,034; adjusted hazard ratio 0.96, 95 % CI 0.75 to 1.23; RD 4 fewer, 95 % CI 25 fewer to 23 more per 1000 over 5 years) and a case–control study provided very low quality evidence also suggesting no association (GLP-1 agonists vs. other anti-hyperglycemic drugs: 1118 cases and 17,626 controls, adjusted OR 0.67, 95 % CI 0.32 to 1.42).

**Conclusions:**

The current evidence suggests that GLP-1 agonists do not increase the risk of heart failure or hospitalization for heart failure among patients with type 2 diabetes.

**Electronic supplementary material:**

The online version of this article (doi:10.1186/s12872-016-0260-0) contains supplementary material, which is available to authorized users.

## Background

Glucagon-like peptide-1 (GLP-1) receptor agonists are a relatively new class of incretin-based agents for the treatment of type 2 diabetes mellitus that lower blood glucose [1, 2], reduce body weight [3], and possibly reduce cardiovascular risk compared to placebo [4, 5]. The American Diabetes Association and the European Association for the Study of Diabetes recommend GLP-1 agonists as a second-line treatment option for type 2 diabetes [6].

In 2014, the US Food and Drug Administration raised concerns regarding heart failure risk with one dipeptidyl peptidase-4 (DPP-4) inhibitor, saxagliptin [7]. These concerns followed publication of studies that reported increased risk of hospitalization for heart failure in patients using DPP-4 inhibitors [8–10]. These observations raise the possibility that GLP-1 agonists, which share a similar pharmacological mechanism with DPP-4 inhibitors, might also cause heart failure.

Animal studies have shown that the GLP-1 agonist liraglutide can activate cytoprotective pathways in the heart, and improve outcomes after experimental myocardial infarction in mice [11]. Early clinical studies also suggested that GLP-1 agonists have positive effects on cardiovascular biomarkers, such as high-sensitivity C-reactive protein and plasminogen activator inhibitor-1 [12, 13], and improve regional and overall left ventricular function in patients with acute myocardial infarction and severe systolic dysfunction after successful primary angioplasty [14].

Clinical trial results often, however, prove inconsistent with laboratory and surrogate outcome studies, and emerging randomized trials and observational studies have, reported inconsistent results [15–19]. We therefore undertook a systematic review to address the effect of GLP-1 agonists on heart failure or hospitalization for heart failure in patients with type 2 diabetes.

## Methods

We followed the PRISMA and MOOSE guidelines for conducting and reporting systematic reviews and meta-analyses of randomized controlled trials (RCTs) and observational studies [20, 21].

### Data sources and search strategy

We searched MEDLINE, EMBASE, and the Cochrane Central Register of Controlled Trials (CENTRAL) from inception to 25 June, 2015. We used both MeSH and free text terms to identify relevant articles. An information expert (DP) developed each database-specific search strategy (Additional file 1). We also searched ClinicalTrials.gov as well as conference abstracts published by the American Diabetes Association, European Association for the Study of Diabetes, and European Society of Cardiology for additional eligible studies and trial information.

### Eligibility criteria

We included RCTs, cohort studies, or case–control studies that compared GLP-1 agonists against placebo, lifestyle modification, or active anti-hyperglycemic medication in adult type 2 diabetes patients, reported ≥ 12 weeks follow-up data (not applicable to case–control studies), and explicitly reported the outcome of heart failure or hospitalization for heart failure.

### Study selection

Paired reviewers, trained in research methods, independently screened titles/abstracts and then full texts for eligibility, assessed risk of bias, and collected data from each included study, using pilot-tested standardized forms with corresponding detailed instructions. Any disagreement between the two reviewers was resolved through discussion or adjudication by a third reviewer (XS).

### Risk of bias and quality of evidence assessment

We assessed the risk of bias of RCTs according to modified version of the Cochrane Collaboration’s tool [22, 23] in which the response options are "probably yes" and "probably no" instead of "unclear"; the approach has shown to be reliable and valid for blinding [24]. The items include randomization sequence generation; allocation concealment; blinding of participants, caregivers, outcome assessors (i.e., heart failure or hospitalization for heart failure), and outcome adjudicators; prognostic balance between treatment groups; and incomplete outcome data.

We used a modified version of the Newcastle – Ottawa Quality Assessment Scale [25–27] for assessing risk of bias of observational studies. Specifically, we removed two items “representativeness of the exposed cohort” and “was follow-up long enough for outcomes to occur” that we judge related to applicability, and added two items - ascertainment of type 2 diabetes and adjustment for potential confounding factors. We planned to assess for risk of publication bias, but were unable to do so due to low power of the relevant tests in the presence of low events rates.

We rated the quality of evidence for heart failure and hospitalization for heart failure as high, moderate, low, or very low using the Grading of Recommendations Assessment, Development and Evaluation (GRADE) methodology [28–34].

### Data extraction

We collected the following information from each eligible studies: study characteristics (e.g., author name, year of publication, study design, sample size, length of follow-up), patient characteristics (e.g., gender, age, diabetes duration, body mass index (BMI), baseline HbA1c level), interventions (e.g., details of GLP-1 agonists therapy and control group), and outcomes (number of events and patients included for analyses in each group, as well as adjusted data if available). For trials with multiple reports, we collated all data into a single study [35]; for trials with reports both from ClincialTrials.gov and journal publications, we carefully checked the data for consistency; for trials reporting outcome data of multiple follow up points, we used the data with longest follow up. For observational studies, we also collected information on data source, methods used to control confounding, and reported adjustment factors.

### Statistical analysis

We analyzed RCTs and observational studies separately. We did not combine the outcomes of heart failure and hospitalization for heart failure, as hospitalization for heart failure is likely more serious and of greater importance to patients than heart failure not requiring hospitalization.

We assessed statistical heterogeneity with the Cochran chi-square test and I-squared statistic. We used Peto’s method to pool data from RCTs [36, 37] using random effects models and reported pooled Peto odds ratios (ORs) and associated 95 % confidence intervals (CIs). We conducted four a priori subgroup analyses to explore heterogeneity associated with our pooled estimates: (1) type of control (placebo vs. active treatment), (2) length of follow up (52 weeks or shorter vs. over 52 weeks), (3) mode of therapy (GLP-1 agonists monotherapy vs. add-on/combination therapy), and (4) individual GLP-1 agonists agents (different GLP-1 agonists agents vs. control). We also carried out sensitivity analyses to explore the robustness of our findings using different effect measures, pooling methods, and statistical models.

We pooled adjusted estimates of heart failure from cohort studies using random effects model due to significant variations in the comparison and patient populations among eligible studies.

#### Ethics

Ethical approval was not necessary as this study is a Systematic Review and Meta-Analysis.

## Results

### Study selection

**Fig. 1:**
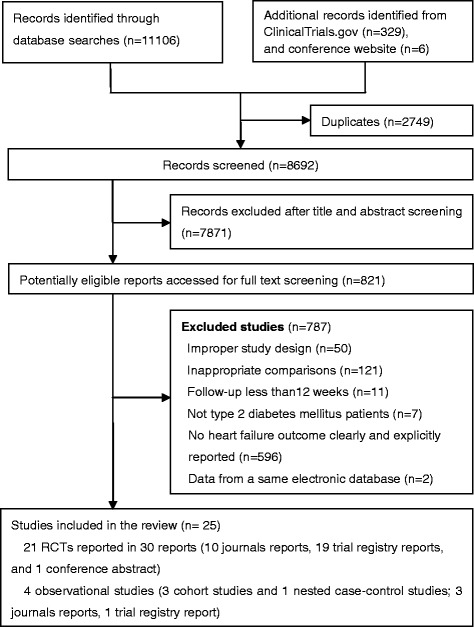
Flow chart of article selection

Our literature search yielded 11,441 reports; 821 were potentially eligible after title and abstract screening, and 25 studies proved eligible after full text screening. These included 21 RCTs involving 18,270 patients from 30 reports [15, 16, 38–65] and four observational studies [17–19, 66] involving 111,029 patients (three cohort studies and one nested case–control study) (Fig. 1).

### Evidence from randomized controlled trials

#### RCTs reporting heart failure

Twenty trials reported on heart failure; 18 (80 %) were multi-center studies, and 18 (90 %) were clearly labeled as phase III trials. These trials enrolled 46 to 1,091 patients (total 12,199); the mean age of patients ranged 52.9 to 67.2 years old, mean BMI 25.6 to 33.3 kg/m^2^, mean baseline HbA1c 7.6 to 8.5 %, mean FPG 7.1 to 10.0 mmol/L, and mean or median duration of diabetes was 2.6 to 11.5 years (Table 1). Five used GLP-1 agonists as monotherapy, 15 as add-on or combination therapy (Table 2). The length of follow-up ranged from 16 to 164 weeks (median 52; 10 trials followed patients for > 52 weeks).

**Table 1:** Baseline characteristics of included randomized controlled trials

Study	International study	Number of countries involved	Number of study sites	Study phase	Total number of patients randomized	Length of follow up (weeks)	Male (*n*,%)	Mean age (years)	Mean BMI (kg/m^2^)	Mean HbA1c (%)	Mean FPG (mmol/L)	Mean diabetes duration (years)
Trials reporting heart failure												
Inagaki 2012 [38, 39]	No	1	NR	III	427	26	290 (67.9)	56.8	26.1	8.5	NR	9.0
NCT00294723 2010 [40, 41]	Yes	2	138	III	746	104	371 (49.7)	53.0	33.1	8.3	9.4	5.4
NCT00318461 2010 [42–44]	Yes	21	170	III	1091	104	635 (58.2)	56.7	31.0	8.4	10.0	7.6
NCT00360334 2009 [45]	No	1	35	III	235	26	160 (68.4)	56.6	NR	NR	NR	6.0
NCT00614120 2010 [46]	Yes	3	51	III	929	16	514 (55.3)	53.3	25.6	NR	NR	7.5
NCT00701935 2013 [47]	Yes	2	17	II	80	26	42 (52.5)	58.1	NR	NR	NR	NR
NCT00838903 2014 [48, 49]	Yes	10	289	III	1049	164	482 (47.6)	54.5	32.6	8.1	9.2	6.0
NCT00838916 2014 [50, 51]	Yes	4	222	III	779	164	418 (56.1)	55.5	33.1	8.3	9.5	8.8
NCT00839527 2014 [52]	Yes	9	358	III	685	164	353 (53.2)	55.2	NR	NR	NR	NR
NCT00849017 2014 [53]	Yes	3	262	III	309	164	166 (55.1)	52.9	NR	NR	NR	NR
NCT00849056 2014 [54]	Yes	6	331	III	310	156	180 (59.8)	55.0	NR	NR	NR	NR
NCT00855439 2015 [55]	No	1	1	NR	46	82	26 (56.5)	53.0	NR	NR	NR	NR
NCT00960661 2013 [56, 57]	Yes	17	108	III	637	30	261 (51.2)	59.5	32.5	8.2	7.1	11.5^a^
NCT01064687 2015 [58]	Yes	3	89	III	978	26	570 (58.4)	55.7	33.2	8.1	9.0	8.8
NCT01075282 2015 [59]	Yes	20	78	III	810	78	353 (51.3)	56.7	31.6	8.1	9.1	9.1
NCT01126580 2015 [60, 61]	Yes	19	91	III	807	56	353 (43.7)	55.6	33.3	7.6	9.0	2.6
NCT01191268 2014 [62]	Yes	16	101	III	884	52	473 (53.5)	59.4	32.5	8.5	NR	12.7
NCT01512108 2014 [63]	No	1	36	III	363	52	262 (72.8)	59.5	NR	8.1	8.8	NR
NCT01620489 2014 [64]	Yes	6	50	III	277	26	140 (50.5)	67.2	NR	NR	NR	NR
Pratley 2013 [65]	Yes	17	130	III	760	24	362 (48.9)	56.4	32.7	8.3	10.0	8.8
Trials reporting hospitalization for heart failure												
Bentley-Lewis 2015 (ELIXA) [15, 16]	Yes	49	NR	III	6068	108^b^	4207 (69.3)	60.3	30.2	7.7	8.2	9.3

*BMI* body mass index, *FPG* fasting plasma glucose, *NR* not reported

^a^median diabetes duration (years); ^b^median follow up time (weeks)

**Table 2:** Intervention tested and event rates in randomized controlled trials

Study	Medications used across groups	Incretin		Control		Duration of treatment (weeks)
		Type	Events	Type	Events	
Trials reporting heart failure						
Inagaki 2012 [38, 39]	BG or BG + TZD	Exenatide	1/215	Insulin glargine	0/212	26
NCT00294723 2010 [40, 41]	None	Liraglutide	1/497	Glimepiride	0/248	104
NCT00318461 2010 [42–44]	Metformin	Liraglutide	1/724	Placebo	0/121	104
		Liraglutide	1/724	Glimepiride	0/242	
NCT00360334 2009 [45]	OADs	Exenatide	0/118	Insulin glargine	1/116	26
NCT00614120 2010 [46]	Merformin	Liraglutide	1/697	Glimepiride	0/231	16
NCT00701935 2013 [47]	None	Exenatide	0/43	Placebo	1/37	26
NCT00838903 2014 [48, 49]	Metformin	Albiglutide	2/302	Placebo	0/101	156
		Albiglutide	2/302	Glimepiride	1/307	
NCT00838916 2014 [50, 51]	Metformin ± SU	Albiglutide	2/504	Insulin glargine	2/241	156
NCT00839527 2014 [52]	Metformin + glimepiride	Albiglutide	0/271	Placebo	1/115	164
		Albiglutide	0/271	Pioglitazone	4/277	
NCT00849017 2014 [53]	None	Albiglutide	1/200	Placebo	2/101	164
NCT00849056 2014 [54]	Pioglitazone ± Metformin	Albiglutide	0/150	Placebo	1/151	156
NCT00855439 2015 [55]	Other diabetes medications	Exenatide	1/22	Glargine	1/24	78
NCT00960661 2013 [56, 57]	Insulin glargine + metformin	Exenatide	0/315	Insulin lispro	1/312	30
NCT01064687 2015 [58]	Metformin and pioglitazone	Dulaglutide	1/559	Placebo	0/141	26
		Exenatide	0/278	Placebo	0/141	
NCT01075282 2015 [59]	Metformin and glimepiride	Dulaglutide	3/545	Insulin glargine	1/262	78
NCT01126580 2015 [60, 61]	None	Dulaglutide	1/539	Metformin	0/268	52
NCT01191268 2014 [62]	Insulin lispro	Dulaglutide	0/588	Insulin glargine	1/296	52
NCT01512108 2014 [63]	None	Liraglutide	1/240	Additional OAD	0/120	52
NCT01620489 2014 [64]	OAD and/or insulin	Liraglutide	1/140	Placebo	0/137	26
Pratley 2013 [65]	SU ± metformin	Taspoglutide	0/494	Pioglitazone	2/257	24
Trials reporting hospitalization for heart failure						
Bentley-Lewis 2015 (ELIXA) [15, 16]	Metformin, SU, glinide, TZD, insulin, metformin and SU, insulin and OADs, or other diabetes medications	Lixisenatide	122/3034	Placebo	127/3034	100

*BG* biguanide, *TZD* thiazolidinedione, *OADs* oral antidiabetic drugs, *SU* sulfonylurea

All the trials reported industry funding; 18 were identified from ClinicalTrials.gov, of which 12 had no corresponding journal publications. Because of the limited information provided in the trial registry, we were unable to adequately assess the risk of bias for these 12 trials. Additional file 2 presents the details of the assessment for risk of bias. The baseline demographics and clinical characteristics of patients in each included trials were generally balanced between groups. The overall risk bias of eligible RCTs was moderate.

**Fig. 2:**
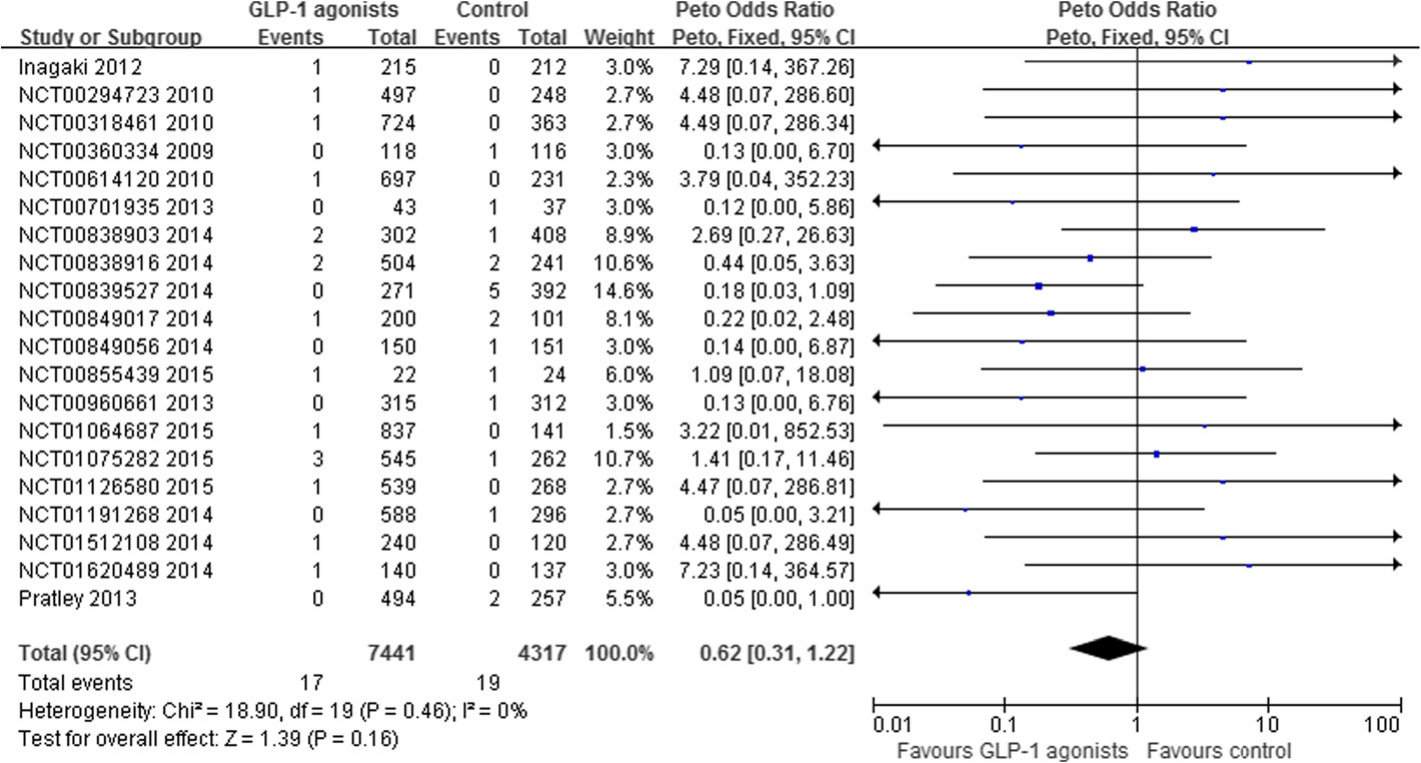
Risk of heart failure in patients who received GLP-1 agonists versus control from randomized controlled trials

Twenty trials reported 36 heart failure events in 11,758 patients using at least one medication (raw event rate 0.3 %). The pooling of those trials showed no statistically significant difference in the risk of heart failure between GLP-1 agonists treatment and control (17/7,441 in GLP-1 agonists and 19/4,317 control; OR 0.62, 95 % CI 0.31 to 1.22, I-square = 0 %; risk difference (RD) 19 fewer, 95 % CI 34 fewer to 11 more per 1000 over 5 years) (Fig. 2). We rated the quality of evidence as low because of risk of bias and imprecision (Table 3).

**Table 3:** GRADE evidence profile of glucagon-like peptide-1 receptor agonists and risk of heart failure in type 2 diabetes

Quality assessment						Summary of findings					Quality of evidence
						Study event rates		Relative risk (95 % CI)	Anticipated absolute effects (5-year time frame)		
No of participants (studies) Follow-up time	Risk of bias	Inconsistency	Indirectness	Imprecision	Publication bias	With control	With GLP-1 agonists		Risk with control	Risk difference with GLP-1 agonists (95 % CI)	
Heart failure											
11758 (20) 16-164 weeks	Serious limitation due to risk of bias^a^	No serious limitations	No serious limitations	Serious limitation, confidence interval includes important benefit and harm	Undetected	19/4317 (0.44 %)	17/7441 (0.23 %)	OR 0.62 (0.31 to 1.22)	50 per 1000^b^	19 fewer per 1000 (34 fewer to 11 more)	⊕ ⊕ ΟΟ **Low** due to risk of bias and imprecision
Hospitalization for heart failure											
6068 (1) 2.1 years	No serious limitations	No serious limitations	No serious limitations	Serious limitation, confidence interval includes important benefit and harm	Undetected	127/3034 4.2 %	122/3034 4 %	HR 0.96 (0.75 to 1.23)	100 per 1000^c^	4 fewer per 1000 (25 fewer to 23 more)	⊕ ⊕ ⊕Ο **Moderate** due to imprecision

*GLP-1* glucagon-like peptide-1

^a^Several trials probably had risk of bias on random sequence generation, allocation concealment and blinding (Additional file 2), and the follow up (median of 52 weeks) was not long enough for heart failure to occur in patients with low risk of cardiovascular disease

^b^Baseline risk estimate for heart failure in a 5-year time frame comes from the control arm of the cohort study we identified to best represent our target population (Kannan 2015 [17]), with 528 events of heart failure in 13,185 participants (4.0 %) at four year follow up across control and intervention arm

^c^Baseline risk estimate for hospitalization for heart failure in 5-year time frame comes from the control arm of the only included ELIXA trial [16] we identified to best represent our target population with 127 events in 3034 participants (42 per 1000) over a 2.1 year follow up period, in the absence of observational studies providing more credible baseline risk estimates

Subgroup analysis by type of control (interaction *p* = 0.79), mode of therapy (interaction *p* = 0.84) and length of follow up (interaction *p* = 0.64) showed no differential treatment effects (Additional files 3, 4, 5 and 6). The subgroup analysis of heart failure risk by individual GLP-1 agonists agents suggested a possibility of differential treatment effect across individual agents (interaction *p* = 0.07), with liraglutide associated with a non-significant increased risk for heart failure (OR 4.85, 95 % CI 0.75 to 31.36); this finding was however based on a limited number of events (five in total) and characterized with very wide confidence interval.

Sensitivity analysis using alternative effect measures, statistical methods, and analysis models did not show important changes in pooled effects.

#### Trials reporting hospitalization for heart failure

The Evaluation of LIXisenatide in Acute Coronary Syndrome (ELIXA) trial, designed to assess the cardiovascular safety of lixisenatide, reported hospitalization for heart failure [15, 16] (Table 1). The ELIXA trial randomized 6,068 patients with type 2 diabetes and a recent acute coronary syndrome to lixisenatide or placebo, with a median of follow up of 2.1 years. In this trial, 122 patients were hospitalized for heart failure among 3,034 patients taking lixisenatide (4.0 %) and 127 in 3034 patients taking placebo (4.2 %), and no statistically significant difference was present between the groups (hazard ratio (HR) 0.96, 95 % CI 0.75 to 1.23; RD 4 fewer, 95 % CI 25 fewer to 23 more per 1000 over 5 years). The trial authors' subgroup analysis by type of history of heart failure showed no differential treatment effects (lixisenatide vs. placebo: patients with history of heart failure: HR 0.93, 95%CI 0.66 to 1.30; patients with no history of heart failure: HR 0.97, 95 % CI 0.67 to 1.40). We rated the quality of evidence as moderate (Table 3).

### Evidence from observational studies

#### Studies reporting heart failure

Three cohort studies [17, 18, 66] reported heart failure. Of these, one prospectively designed study [66] examined exenatide versus basal insulin; the other two [17, 18] – retrospective in design - assessed GLP-1 agonists versus sulfonylureas, and exenatide or exenatide plus insulin versus insulin (Tables 4 and 5). The sample sizes ranged from 882 to 39,225, and length of follow up ranged from 1 to 4 years. The mean age ranged from 58.28 to 62.5 years, BMI 32.6 to 35.3 kg/m^2^, and mean baseline HbA1c 7.9 to 8.9 %.

**Table 4:** Characteristics of included observational studies

Study	Study design	Data source	Countries involved	Funding	Total number of patients	Follow up (years)	Male (*n*, %)	Mean age (years)	Mean BMI (kg/m^2^)	Mean HbA1c (%)	Mean FPG (mmol/L)	Mean diabetes duration (years)	CVD at baseline
Studies reporting heart failure													
NCT01060059 2013 [66]	Prospective cohort study	Real world data	Italy	Private for-profit funding	882	1	493 (55.9)	62.5	NR	8.9	NR	NR	NR
Kannan 2015 [17]^a^	Retrospective cohort study	Electronic health records	U.S.	No funding	13,185	4^b^	7827 (54.6)	60.6	32.6^c^	NR	NR	NR	Included patients had no history of CVD or congestive heart failure at baseline
Paul 2015 [18]	Retrospective cohort study	Claims data	U.S.	NR	39,225	3.5^b^	18093 (46.1)	58.2	35.3	7.9	NR	1.3	Included patients had CVD or no CVD at baseline
Studies reporting hospitalization for heart failure													
Yu 2015 [19]^a^	Nested case–control study	Electronic medical records	UK	Public funding	57,737	NA	32795 (56.8)	61.6	NR	NR	NR	2.3	Included patients had CVD or no CVD at baseline

^a^These two studies accessed incretin agents (both glucagon-like peptide-1 receptor agonists and dipeptidyl peptidase-4 inhibitors) and the risk of heart failure, so the data above were the characteristics of total patients included

*BMI* body mass index, *FPG* fasting plasma glucose, *CVD* cardiovascular disease, *NR* not reported, *NA* not applicable

^b^median follow-up (years); ^c^Median BMI (kg/m^2^)

**Table 5:** Exposures, outcomes, and results of observational studies

Study	Exposure of interest	Control group	Number of events or cases	Total number of analyzed patients	Adjusted estimates (95 % CI)	Adjusted covariate
Studies reporting heart failure						
Kannan 2015 [17]	GPL-1 agonists (combined with metformine)	Sulfonylureas (combined with metformine)	528^a^	13,185 (55,110 person years)^a^	HR 1.10 (0.99 to 1.22)	Age, sex, race, BMI, number of encounters, median household income, smoking status, systolic and diastolic blood pressure, hypertension, dyslipidemia, cerebral vascular event, presence of neuropathy, retinopathy, dementia, chronic obstructive pulmonary disease, cancer, atrial fibrillation, anti-hypertensive medications, lipid lowering agents, anti-platelet agents and propensity for being on metformin and sulfonylureas at baseline, lipid profile, estimated glomerular filtration rate
Paul 2015 [18]	Exenatide/exenatide + insulin	Insulin	2338	39,225	Exenatide vs insulin: HR 0.34 (0.22, 0.52) Exenatide + insulin vs insulin: HR 0.40 (0.32, 0.50) Without previous CVD: Exenatide vs insulin: HR 0.34 (0.22, 0.52) Exenatide + insulin vs insulin: HR 0.40 (0.32, 0.50) Without previous CVD & renal diseases: Exenatide vs insulin: HR 0.32 (0.21, 0.50) Exenatide + insulin vs insulin: HR 0.35 (0.28, 0.45)	Gender, ethnicity, age at the start of cohort, BMI, HbA1c, systolic and diastolic blood pressure on the index date, history of cardiovascular disease, any renal disease prior to index date or during follow-up, use of metformin, sulfonylurea, cardioprotective medications or antihypertensive medications, and the duration of diabetes
NCT01060059 2013 [66]	Exenatide	Basal insulin	2	882	NR	NR
Studies reporting hospitalization for heart failure						
Yu 2015 [19]	GLP-1 agonists (exenatide and liraglutide, alone or incombination with other antidiabetic drugs)	Other oral antidiabetic drugs	1,118^a^	18,744^a^	OR 0.67 (0.32 to 1.42)	Sex, BMI, excessive alcohol use, smoking status, HbA1c level, comorbidities (neuropathy, renal disease, retinopathy, atrial fibrillation, cancer [other than nonmelanoma skin cancer], chronic obstructive pulmonary disease, coronary artery disease, dyslipidemia, hypertension, previous myocardial infarction, peripheral arteriopathy, previous coronary revascularization, peripheral vascular disease, and previous stroke), number of prescriptions, number of physician visits, and use of the following drugs in the year prior to cohort entry: angiotensin converting enzyme inhibitors, angiotensin receptor blockers, β-blockers, calcium channel blockers, diuretics, fibrates, statins, aspirin, and other nonsteroidal anti-inflammatory drugs

^a^These two studies accessed incretin agents and the risk of heart failure, and the data of events/cases and total number of analyzed patients regarding glucagon-like peptide-1 receptor agonists and dipeptidyl peptidase-4 inhibitors were not reported separately, so the data above were the data of total study patients

*CI* confidence interval, *NR* not reported, *HR* hazard ratio, *OR* odds ratio, *CVD* cardiovascular disease, *BMI* body mass index

The three studies used electronic heath records or claims data for their analyses. Type 2 diabetes patients were ascertained by specialists in outpatient setting in the prospective cohort study [66]; the other two retrospective cohort study [17, 18] did not explicitly state the ascertainment of type 2 diabetes. None of these studies mentioned the ascertainment of exposure to GLP-1 agonist agents and other confounding variables. Only one study [17] demonstrated that outcome of interest was not present at start of study, and mentioned the method used to assess the outcome of interest. Two studies [18, 19] used advanced statistical model to control for the influence of confounding factors. Overall, the risk of bias associated with these studies was moderate to high (Additional file 7).

**Fig. 3:**
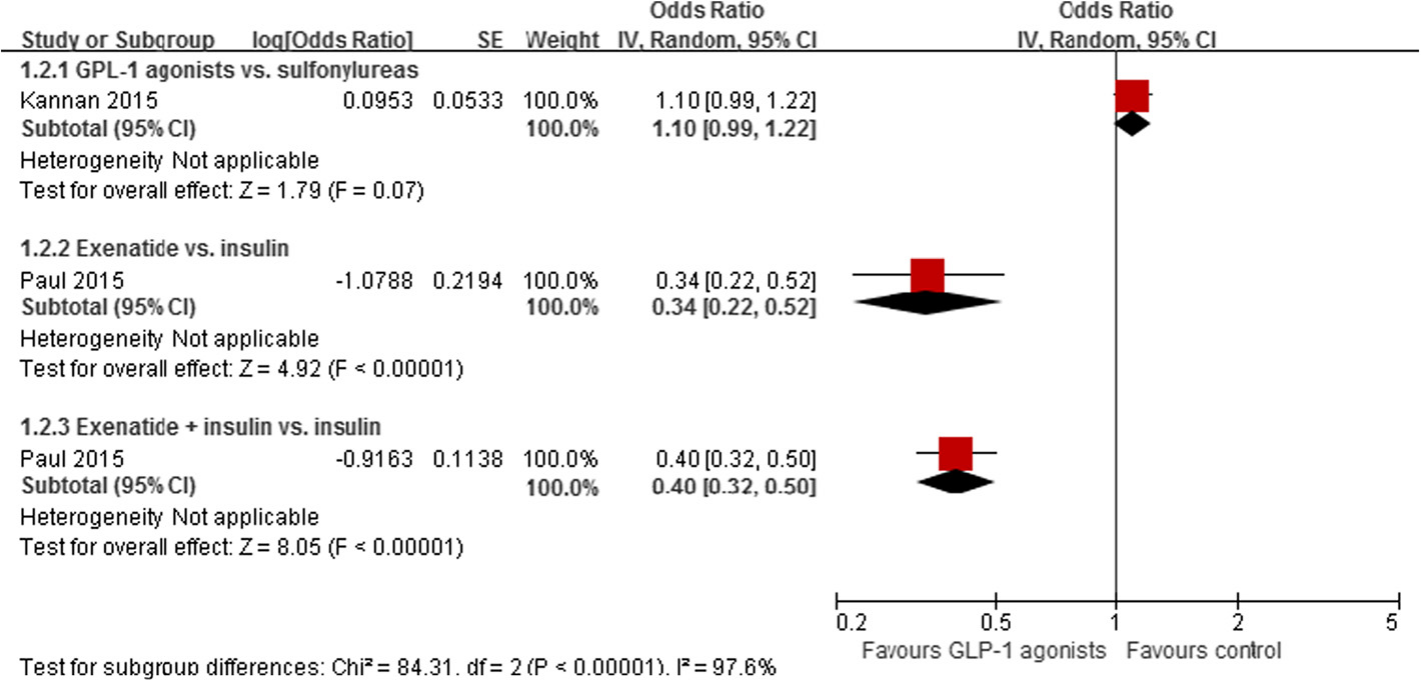
Risk of heart failure in patients who received GLP-1 agonists versus control based on adjusted data of observational studies

All three studies reported raw data, for a total of 2,868 heart failures among 53,292 patients (raw event rate 5.4 %); two retrospective cohort studies [17, 18] reported adjusted effect estimates (Tables 5 and 6). The prospective cohort study [66], enrolling 882 patients with one year follow-up, found that two patients (2/438) in the basal insulin had heart failure events and no patients (0/444) in exenatide group. One retrospective cohort study [17], including 13,185 patients and with a median follow-up of four years, reported that GLP-1 agonists were associated with a non-significant increase in heart failure versus sulfonylureas (adjusted HR 1.10, 95 % CI 0.99 to 1.22). The other retrospective cohort study [18], involving 39,225 patients and with a median follow-up of 3.5 years, found that both exenatide and exenatide plus insulin were associated with a lower risk of heart failure versus insulin alone (adjusted HR 0.34, 95 % CI 0.22 to 0.52; adjusted HR 0.40, 95 % CI 0.32 to 0.50, respectively, Fig. 3). Using GRADE, we rated the quality of evidence in the included studies as very low, due to risk of bias, indirectness and heterogeneity in addition to the inherent risk for confounding associated with observational studies.

**Table 6:** Risk of heart failure or hospitalization for heart failure among patients with type 2 diabetes receiving glucagon-like peptide-1 receptor agonists treatment

Comparison	Number of studies (Events or cases, patients)	GLP-1 agonists (events/patients)	Control (events/patients)	Effect Estimate (95%CI)	Cardiovascular morbidities at baseline
1. Heart failure					
Randomized controlled trials					
GLP-1 agonists vs. control	20 (36, 11758)	17/7441	19/4317	Pooled OR 0.62 (0.31 to 1.22)	Typically without CVD at baseline
Cohort studies					
GLP-1 agonists vs. SU	1 (528, 13185)	NR	NR	Adjusted HR 1.10 (0.99 to 1.22)	No history of CVD or congestive heart failure at baseline
Exenatide vs. insulin Exenatide + insulin vs. insulin	1 (2338, 39225)	49/2804 195/7870	2094/28551 2094/28551	Adjusted HR 0.34 (0.22, 0.52) Adjusted HR 0.40 (0.32, 0.50)	With or without CVD at baseline
Exenatide vs. basal insulin	1 (2, 882)	0/444	2/438	Unadjusted OR 0.13 (0.01 to 2.13)	NR
2. Hospitalization for heart failure					
Randomized controlled trials					
Lixisenatide vs. placebo	1 (249, 6068)	122/3034	127/3034	Pooled Adjusted HR 0.96 (0.75, 1.23)	Acute coronary syndrome
Nested case–control studies					
GLP-1 agonists vs. other OADs	1 (1118, 18744)			Adjusted OR 0.67 (0.32 to 1.42)	With or without CVD at baseline

*GLP-1* glucagon-like peptide-1, *CVD* cardiovascular disease, *SU* sulfonylurea, *OR* odds ratio, *HR* hazard ratio, *NR* not reported, *OADs* oral antidiabetic drugs

#### Studies reporting hospitalization for heart failure

One nested case–control study [19] assessed with GLP-1 agonists versus other oral anti-hyperglycemic drugs (Tables 4 and 5). This study included 57,737 patients, with a mean age of 61.6 years and mean duration of diabetes 2.3 years. The methodological details regarding the control for bias are provided in Additional file 8. This study included 1118 cases and 17,626 matched controls and found that, compared to the use of other anti-hyperglycemic drugs, GLP-1agonists were not associated with increased risk of hospitalization for congestive heart failure (adjusted OR 0.67, 95 % CI 0.32 to 1.42). Using GRADE, we rated the quality of evidence as very low, due to risk of bias and imprecision in addition to the inherent risk for confounding associated with observational studies.

## Discussion

### Main findings

Our pooled analysis of 20 RCTs addressing use of GLP-1 agonists for type 2 diabetes found moderate quality evidence suggesting no increase in heart failure. The only RCT provided high quality evidence that lixisenatide did not increase the risk of hospitalization due to heart failure. Though the four observational studies provide only very low quality evidence, their results are consistent with those from the randomized trials.

### Strengths and limitations

We are the first to systematically review the evidence regarding GLP-1 agonists for type 2 diabetes and risk of heart failure. Our study has several strengths. First, we used rigorous methods to systematically identify both randomized and observational studies that reported data to inform this issue, including a large number of trials that were not published in journals. Second, we carefully checked the data reported in ClinicalTrials.gov and journal publications for consistency to ensure accuracy of the data. Third, we analysed the data on heart failure and hospitalization for heart failure separately, because those outcomes are likely to be of different importance to patients. Fourth, we used the GRADE approach to assess the quality of evidence on an outcome-by-outcome basis.

Our study also has limitations. First, the available evidence is not strength to give definitive answer for this question, since the included RCTs reported few heart failure events and the follow-up was not enough for heart failure to occur, and much findings came from observational studies of very low quality evidence. Second, we have included some observational studies at moderate to high risk of bias. This has made the inference about the effects of GLP-1 agonists challenging. Third, the diversity of observational studies also made our analysis of the evidence difficult. One retrospective cohort study [18], assessing exenatide and/or insulin on heart failure outcome, included patients with heart failure at baseline, and the proportion of patients with history of heart failure was higher in the insulin group (3.2 %) than in the exenatide group (1.7 %) and exenatide + insulin group (2.4 %), which made the finding from this study biased.

### Other researches

Ours is the first systematic review addressing the impact of GLP-1 agonists on heart failure. There is some evidence from human studies that GLP-1 agonists might provide protection against heart failure: preliminary study [67] showed that GLP-1 treatment might have a trend towards improvement of cardiac function in type 2 diabetes patients with stable heart failure; intrinsic GLP-1 expression has been shown to compensatorily upregulate in patients with left heart failure [68]; and GLP-1 agonists are also shown to be associated with a modest increase of ejection fraction in diabetic patients [69]. A recent meta-analysis of RCTs [70] found that GLP-1 agonists were associated with a modest reduction in blood pressure and a slight increase in heart rate. These biological studies suggest that GLP-1 agonists might, if anything, reduce the incidence of heart failure. Though results of RCTs fail to show this decrease, confidence intervals do not exclude the possibility of a modest reduction.

## Conclusions

The current evidence suggests that GLP-1 agonists do not increase the risk of heart failure or hospitalization for heart failure. The current body of evidence, however, is not definitive. More carefully designed, conducted, adequately powered trials and observational studies are warranted to confirm the effects of GLP-1 agonists on incidence of heart failure and hospitalization for heart failure. Future studies should also examine whether the effects of GLP-1 agonists on heart failure are affected by patient's baseline risk of cardiovascular disease.

## Availability of data and materials

The datasets supporting the conclusions of this article are included within the article and its additional files.

## Additional files

Additional file 1: Search strategies. (DOC 35 kb) Additional file 2: Risk of bias of included randomized controlled trials. (DOC 78 kb) Additional file 3: Subgroup analysis of heart failure risk by type of control based on raw data of randomized controlled trials. (DOC 47 kb) Additional file 4: Subgroup analysis of heart failure risk by mode of therapy based on raw data of randomized controlled trials. (DOC 45 kb) Additional file 5: Subgroup analysis of heart failure risk by length of follow up based on raw data of randomized controlled trials. (DOC 45 kb) Additional file 6: Subgroup analysis of heart failure risk by individual GLP-1 agonists agents based on raw data of randomized controlled trials. (DOC 55 kb) Additional file 7: Risk of bias of included cohort studies. (DOC 55 kb) Additional file 8: Risk of bias of included case–control studies. (DOC 52 kb)

## Abbreviations

BMI: body mass index
CENTRAL: the Cochrane Central Register of Controlled Trials
CI: confidence interval
DPP-4: dipeptidyl peptidase-4
ELIXA: Evaluation of LIXisenatide in Acute Coronary Syndrome
FPG: fasting plasma glucose
GLP-1: glucagon-like peptide-1
GRADE: Grading of Recommendations Assessment, Development and Evaluation
HbA1c: glycated haemoglobin
HR: hazard ratio
MOOSE: Meta-analysis Of Observational Studies in Epidemiology
OR: odds ratio
PRISMA: Preferred Reporting Items for Systematic Reviews and Meta-Analyses
RCTs: randomized controlled trials
RD: risk difference
