# Supplementary material for: Glucagon-like peptide-1 receptor agonists and heart failure in type 2 diabetes: systematic review and meta-analysis of randomized and observational studies
Source: BMC Cardiovasc Disord. 2016 May 11;16:91. doi: 10.1186/s12872-016-0260-0 (PMC4863354; doi:10.1186/s12872-016-0260-0)
Supplement: Additional file 1: — Search strategies. (DOC 35 kb) [file 12872_2016_260_MOESM1_ESM.doc]

**Additional file 1 Search strategies**

In our search, we included terms that defined incretin drugs (glucagon-like peptide-1 receptor agonists and dipeptidyl peptidase-4 inhibitors) and study designs, because the greater study was planned to assess the all aspects regarding safety of incretin therapies on diabetic and non-diabetic populations.

1. **MEDLINE (Ovid)** **(**Search date: June 25, 2015**)**

1 glucagon like peptide*.ti,ab.

2 Receptors, Glucagon/ag [Agonists]

3 exenatide.af.

4 liraglutide.af.

5 albiglutide.af.

6 taspoglutide.af.

7 lixisenatide.af.

8 dulaglutide.af.

9 semaglutide.af.

10 ZP10A peptide*.af.

11 GLP 1 Receptor Agonist*.af.

12 GLP 1 RA*.af.

13 GLP 1RA*.af.

14 dipeptidyl peptidase IV inhibit*.af.

15 Dipeptidyl Peptidase 4 Inhibit*.af.

16 DPP 4 inhibit*.af.

17 DPP4 inhibit*.af.

18 DPP4i.af.

19 DPP IV inhibit*.af.

20 DPPIV inhibit*.af.

21 alogliptin.af.

22 gemigliptin.af.

23 "LC15 0444".af.

24 linagliptin.af.

25 saxagliptin.af.

26 sitagliptin.af.

27 vildagliptin.af.

28 dutogliptin.af.

29 teneligliptin.af.

30 anagliptin.af

31 evogliptin.af

32 DA-1229.af

33 retagliptin.af

34 gliptin*.af.

35 1 or 2 or 3 or 4 or 5 or 6 or 7 or 8 or 9 or 10 or 11 or 12 or 13 or 14 or 15 or 16 or 17 or 18 or 19 or 20 or 21 or 22 or 23 or 24 or 25 or 26 or 27 or 28 or 29 or 30 or 32 or 33 or 34

36 (clinical trial or controlled clinical trial or randomized controlled trial).pt.

37 clinical trials as topic/ or controlled clinical trials as topic/ or randomized controlled trials as topic/

38 random*.ti,ab.

39 clinical trial*.ti,ab.

40 controlled trial*.ti,ab.

41 case-control studies/

42 retrospective studies/

43 cohort studies/

44 longitudinal studies/

45 follow-up studies/

46 prospective studies/

47 cohort.ti,ab.

48 longitudinal.ti,ab.

49 follow up.ti,ab.

50 followup.ti,ab.

51 prospective*.ti,ab.

52 retrospective*.ti,ab.

53 nonrandom*.ti,ab.

54 comparison group*.ti,ab.

55 control group*.ti,ab.

56 database*.ti,ab.

57 population*.ti,ab.

58 registries/

59 registry.ti,ab.

60 registries.ti,ab.

61 36 or 37 or 38 or 39 or 40 or 41 or 42 or 43 or 44 or 45 or 46 or 47 or 48 or 49 or 50 or 51 or 52 or 53 or 54 or 55 or 56 or 57 or 58 or 59 or 60

62 35 and 61

63 limit 62 to humans

64 limit 62 to animals

65 64 not 63

66 64 not 65

67 meta analysis.pt.

68 Meta-Analysis as Topic/

69 meta analy*.ti.

70 metaanaly*.ti.

71 67 or 68 or 69 or 70

72 35 and 71

73 limit 72 to humans

74 limit 72 to animals

75 74 not 73

76 72 not 75

77 66 not 76

78 remove duplicates from 77

1. **EMBASE (Ovid) (**Search date: June 25, 2015**)**

1 exp glucagon like peptide/

2 exp glucagon like peptide 1/

3 glp-1 receptor agonists.mp.

4 glucagon like peptide 1 receptor agonists.mp.

5 exp receptors, glucagon/

6 exp exenatide/

7 exp liraglutide/

8 exp albiglutide/

9 exp taspoglutide/

10 exp lixisenatide/

11 exp dulaglutide/

12 exp semaglutide/

13 zp10a peptide.mp.

14 zp10a peptide 1.mp.

15 glp 1 ra.mp.

16 dipeptidyl peptidase iv inhibit.mp.

17 exp dipeptidyl peptidase IV inhibitor/

18 exp dipeptidyl peptidase IV inhibitor/

19 dpp 4 inhibitor.mp.

20 dpp4i.mp.

21 dpp iv inhibitor.mp.

22 dppiv inhibitor.mp.

23 exp alogliptin/

24 exp gemigliptin/

25 "lc15 0444".mp.

26 exp linagliptin/

27 exp saxagliptin/

28 exp sitagliptin/

29 exp vildagliptin/

30 exp dutogliptin/

31 exp teneligliptin/

32 exp anagliptin/

33 exp evogliptin/

34 DA-1229.mp

35 retagliptin.mp

36 gliptin.mp.

37 exp clinical trial/ or exp controlled clinical trial/ or exp randomized controlled trial/

38 random*.mp.

39 exp case control study/

40 exp retrospective study/

41 exp cohort analysis/

42 exp longitudinal study/

43 exp follow up/

44 nonrandom.mp.

45 exp prospective study/

46 comparison group.mp.

47 exp control group/

48 database*.mp.

49 exp register/

50 1 or 2 or 3 or 4 or 5 or 6 or 7 or 8 or 9 or 10 or 11 or 12 or 13 or 14 or 15 or 16 or 17 or 18 or 19 or 20 or 21 or 22 or 23 or 24 or 25 or 26 or 27 or 28 or 29 or 30 or 31 or 32 or 33 or 34 or 35 or 36

51 37 or 38 or 39 or 40 or 41 or 42 or 43 or 44 or 45 or 46 or 47 or 48 or 49 or 50

52 50 and 51

53 limit 52 to human

54 limit 52 to animals

55 54 not 53

56 52 not 55

1. **Cochrane Central Register of Controlled Trials** **(Ovid)** (May 2015)

1 glucagon like peptide.mp. or exp Glucagon-Like Peptides/

2 exp Glucagon-Like Peptide 1/ or Glucagon-Like Peptide 1.mp.

3 Receptors, Glucagon/ag [Agonists]

4 exenatide.af.

5 albiglutide.af.

6 taspoglutide.af.

7 lixisenatide.af.

8 dulaglutide.af.

9 liraglutide.af.

10 semaglutide

11 ZP10A peptide*.af.

12 GLP 1 Receptor Agonist*.af.

13 GLP 1 RA*.af.

14 GLP 1RA*.af.

15 dipeptidyl peptidase IV inhibit*.af.

16 Dipeptidyl Peptidase 4 Inhibit*.af.

17 DPP 4 inhibit*.af.

18 DPP4 inhibit*.af.

19 DPP4i.af.

20 DPP IV inhibit*.af.

21 DPPIV inhibit*.af.

22 alogliptin.af.

23 gemigliptin.af.

24 "LC15 0444".af.

25 linagliptin.af.

26 saxagliptin.af.

27 sitagliptin.af.

28 vildagliptin.af.

29 dutogliptin.af.

30 teneligliptin.af.

31 anagliptin.af

32 evogliptin.af

33 DA-1229.af

34 retagliptin.af

35 gliptin*.af.

36 1 or 2 or 3 or 4 or 5 or 6 or 7 or 8 or 9 or 10 or 11 or 12 or 13 or 14 or 15 or 16 or 17 or 18 or 19 or 20 or 21 or 22 or 23 or 24 or 25 or 26 or 27 or 28 or 29 or 30 or 31 or 32 or 33 or 34 or 35

1. **ClinicalTrials.gov** (Search date: June 25, 2015)

exenatide OR liraglutide OR albiglutide OR taspoglutide OR lixisenatide OR dulaglutide OR semaglutide OR sitagliptin OR vildagliptin OR saxagliptin OR linagliptin OR alogliptin OR dutogliptin OR gemigliptin OR teneligliptin OR anagliptin OR evogliptin OR retagliptin | Studies With Results

** When searching ClinicalTrials.gov, we did not use the general terms, such as “glucagon like peptide-1” and “dipeptidyl peptidase-4”, as this registry is designed so that one can capture relevant trials using generic drug names directly. We did not limit the search to completed studies, because we also included “terminated” studies with results.
