# Supplementary material for: Glucagon-like peptide-1 receptor agonists and heart failure in type 2 diabetes: systematic review and meta-analysis of randomized and observational studies
Source: BMC Cardiovasc Disord. 2016 May 11;16:91. doi: 10.1186/s12872-016-0260-0 (PMC4863354; doi:10.1186/s12872-016-0260-0)
Supplement: Additional file 2: — Risk of bias of included randomized controlled trials. (DOC 78 kb) [file 12872_2016_260_MOESM2_ESM.doc]

**Additional file 2: Risk of bias of included randomized controlled trials**

| **Study** | **Adequate randomization sequence generation** | **Adequate allocation concealment** | **Blinding of participants and personnel** | **Blinded assessment** **HF or HHF events** | **HF or HHF outcome adjudicated** | **Blinded HF or HHF outcome adjudication** | **Free of incomplete outcome data** |
| --- | --- | --- | --- | --- | --- | --- | --- |
| **Trials reporting heart failure** |  |  |  |  |  |  |  |
| Inagaki 2012 [38,39] | Definitely yes | Definitely yes | Definitely no | Definitely no | Probably no | Probably no | Definitely yes |
| NCT00294723 2010 [40,41] | Definitely yes | Definitely yes | Definitely yes | Probably yes | Probably no | Probably no | Probably yes |
| NCT00318461 2010 [42-44] | Definitely yes | Definitely yes | Definitely no | Definitely no | Probably no | Probably no | Probably yes |
| NCT00360334 2009 [45] | Probably no | Probably no | Definitely no | Definitely no | Probably no | Probably no | Probably yes |
| NCT00614120 2010 [46] | Probably yes | Probably yes | Definitely yes | Probably yes | Probably no | Probably no | Probably yes |
| NCT00701935 2013 [47] | Probably yes | Probably yes | Definitely yes | Probably yes | Probably no | Probably no | Definitely yes |
| NCT00838903 2014 [48,49] | Probably yes | Probably yes | Definitely yes | Probably yes | Definitely yes | Probably yes | Probably yes |
| NCT00838916 2014 [50,51] | Definitely yes | Definitely yes | Definitely no | Definitely no | Definitely yes | Definitely yes | Probably yes |
| NCT00839527 2014 [52] | Probably yes | Probably yes | Definitely yes | Probably yes | Probably no | Probably no | Probably yes |
| NCT00849017 2014 [53] | Probably yes | Probably yes | Definitely yes | Probably yes | Probably no | Probably no | Probably yes |
| NCT00849056 2014 [54] | Probably yes | Probably yes | Definitely yes | Probably yes | Probably no | Probably no | Probably yes |
| NCT00855439 2015 [55] | Probably no | Probably no | Definitely no | Definitely no | Probably no | Probably no | Definitely yes |
| NCT00960661 2013 [56,57] | Definitely yes | Probably no | Definitely no | Definitely no | Probably no | Probably no | Probably yes |
| NCT01064687 2015 [58] | Probably yes | Probably yes | Definitely yes | Definitely yes | Definitely no | Definitely no | Probably yes |
| NCT01075282 2015 [59] | Probably no | Probably no | Definitely no | Definitely no | Definitely yes | Definitely no | Probably yes |
| NCT01126580 2015 [60,61] | Definitely yes | Definitely yes | Definitely yes | Definitely yes | Probably no | Probably no | Definitely yes |
| NCT01191268 2014 [62] | Probably no | Probably no | Definitely no | Definitely no | Probably no | Probably no | Definitely yes |
| NCT01512108 2014 [63] | Probably no | Probably no | Definitely no | Definitely no | Probably no | Probably no | Probably yes |
| NCT01620489 2014 [64] | Probably yes | Probably yes | Definitely yes | Probably yes | Probably no | Probably no | Probably yes |
| Pratley 2013 [65] | Definitely yes | Definitely yes | Definitely yes | Probably yes | Probably no | Probably no | Probably yes |
| **Trials reporting hospitalization for heart failure** | | | | | | | |
| Bentley-Lewis 2015 (ELIXA) [15,16] | Definitely yes | Definitely yes | Definitely yes | Definitely yes | Definitely yes | Definitely yes | Definitely yes |

HF: heart failure; HHF: hospitalization for heart failure.
