# Supplementary material for: Glucagon-like peptide-1 receptor agonists and heart failure in type 2 diabetes: systematic review and meta-analysis of randomized and observational studies
Source: BMC Cardiovasc Disord. 2016 May 11;16:91. doi: 10.1186/s12872-016-0260-0 (PMC4863354; doi:10.1186/s12872-016-0260-0)
Supplement: Additional file 3: — Subgroup analysis of heart failure risk by type of control based on raw data of randomized controlled trials. (DOC 47 kb) [file 12872_2016_260_MOESM3_ESM.doc]

**
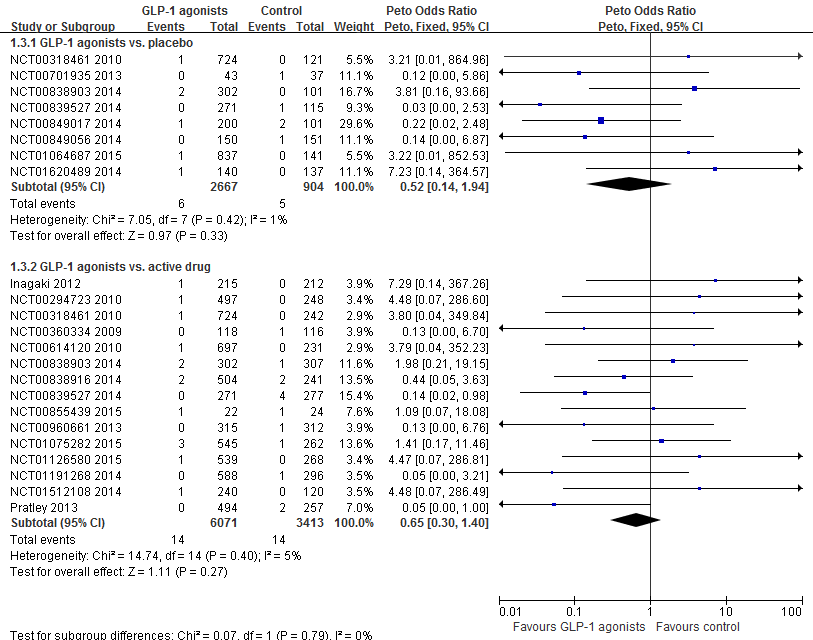
**

**Additional file 3:** Subgroup analysis of heart failure risk by type of control based on raw data of randomized controlled trials
