# Supplementary material for: Glucagon-like peptide-1 receptor agonists and heart failure in type 2 diabetes: systematic review and meta-analysis of randomized and observational studies
Source: BMC Cardiovasc Disord. 2016 May 11;16:91. doi: 10.1186/s12872-016-0260-0 (PMC4863354; doi:10.1186/s12872-016-0260-0)
Supplement: Additional file 7: — Risk of bias of included cohort studies. (DOC 55 kb) [file 12872_2016_260_MOESM7_ESM.doc]

**Additional file 7:** **Risk of bias of included cohort studies**

| **Study** | **Ascertainment of type 2 diabetes conditions** | **Ascertainment of exposure to GLP-1 agonists agents** | **Selection of the non exposed cohort** | **Ascertainment of other** **confounding variables** | **Demonstration that outcome of interest was not present at start of study** | **Comparability of study controls for important factors** | **Assessment of outcome** | **Completeness of outcome and exposure variables** |
| --- | --- | --- | --- | --- | --- | --- | --- | --- |
| **Studies reporting heart failure** | | | | | | | | |
| NCT01060059 2013 [66] | Adult patients with type 2 diabetes mellitus identified and treated in outpatient setting by specialists | Statement not explicit; likely from the data collected during the follow up | Drawn from the same population as the exposed cohort | NR | NR | No, patients had statistical difference in age, weight, and HbA1c, and no adjusted analysis were conducted | Statement not explicit; likely from the data collected during the follow up | Eighteen (4.1%) and 34 (7.8%) in the exenatide and basal insulin were lost to follow-up, respectively |
| Kannan 2015 [17] | Statement not explicit; likely from identifying ICD-9 code in the database | Statement not explicit; likely from the drug prescription in the electronic medical records | Drawn from the same population as the exposed cohort | NR | Yes, patients who had a history of cardiovascular disease or congestive heart failure at baseline were excluded | Cox regression model was used to adjust baseline medical history variables (age, sex, race, BMI, number of encounters, median household income, smoking status, systolic and diastolic blood pressure, drugs used, comorbidities and propensity for being on metformin and sulfonylureas at baseline), and laboratory data (lipid profile, estimated glomerular filtration rate) | ICD-9 codes and/or a documentation of a post-baseline left ventricular ejection fraction < 40% | Authors did not mention the completeness of outcome and exposure variable data in the database |
| Paul 2015 [18] | NR | Statement not explicit; likely from the drug prescription in the electronic medical records | Drawn from the same population as the exposed cohort | NR | No, patients who had a history of heart failure at baseline were included | Cox regression model was used to control for gender, ethnicity, age at the start of cohort, BMI, HbA1c, systolic and diastolic blood pressure on the index date, history of cardiovascular disease, any renal disease prior to index date or during follow-up, use of metformin, sulfonylurea, cardioprotective medications or antihypertensive medications, and the duration of diabetes | Statement not explicit; likely from the ICD-9 code | Included patients had no missing data on age, sex, ethnicity, smoking status, HbA1c at index date, and complete information on event dates for heart failure |

GLP-1= glucagon-like peptide-1; ICD-9= International Classification of Diseases, Ninth Revision; NR= not reported; BMI= body mass index.
