# Supplementary material for: Glucagon-like peptide-1 receptor agonists and heart failure in type 2 diabetes: systematic review and meta-analysis of randomized and observational studies
Source: BMC Cardiovasc Disord. 2016 May 11;16:91. doi: 10.1186/s12872-016-0260-0 (PMC4863354; doi:10.1186/s12872-016-0260-0)
Supplement: Additional file 8: — Risk of bias of included case–control studies. (DOC 52 kb) [file 12872_2016_260_MOESM8_ESM.doc]

**Additional file 8: Risk of bias of included case-control studies**

| Study | Ascertainment of type 2 diabetes conditions | Is case definition adequate | Selection of controls | Definition of controls | Ascertainment of exposure to GLP-1 agonists agents | Ascertainment of other confounding variables | Same method of ascertainment for exposure to GLP-1 agonists agents | Comparability of study controls for important factors | Completeness of data within database |
| --- | --- | --- | --- | --- | --- | --- | --- | --- | --- |
| Studies reporting hospitalization for heart failure | | | | | | | | | |
| Yu 2015 [19] | Statement not explicit; likely from identifying patients newly treated with noninsulin antidiabetic drugs (metformin, sulfonylureas, thiazolidinediones, a-glucosidase inhibitors, guar gum, meglitinides, and incretin-based drugs) | Yes, case subjects were defined by hospitalization for congestive heart failure | Up to 20 control subjects per case were randomly selected using risk set sampling, matched on duration of follow up, age, duration of treated diabetes, and calendar year of study cohort entry | Statement not explicit; likely patients with no hospitalization for congestive heart failure | Statement not explicit; likely from the medical records | Statement not explicit; confounding variables were likely ascertained by identifying the medical records | Statement not explicit; likely from the medical records | In addition to conditioning on the matching variables (i.e., duration of follow-up, age, duration of treated diabetes, and calendar year of cohort entry) , conditional logistic regression was used to control for sex, BMI, excessive alcohol use, smoking status, HbA1c level, comorbidities such as neuropathy, renal disease, retinopathy, atrial fibrillation, cancer, chronic obstructive pulmonary disease, coronary artery disease, dyslipidemia, hypertension, previous myocardial infarction, peripheral arteriopathy, previous coronary revascularization, peripheral vascular disease, and previous stroke, all measured at any time prior to cohort entry | Completeness of data in the  database not  mentioned, however, the database has been shown to be valid and of high quality |

GLP-1= glucagon-like peptide-1; NR= not reported.
